# On the Importance of the Nervous System in the Animal Economy

**Published:** 1815-03

**Authors:** P. P. James

**Affiliations:** Surgeon. *Hereford*


*For the Medical and Physical Journal.*

### *On the Importance of the Nervous System in the Animal Economy;* by Mr. James, of Hereford.

I BEG permission for a small space of your Journal, for a few observations to shew the great importance the nervous system holds in the animal economy, as influencing many intractable and distressing diseases, from alteration in their structure.

During the short time I was in Edinburgh, my attention was directed to a peculiar state of the nerves, by what I heard Dr. Sanders say in his Lectures on the Practice of Medicine—(lectures, which I shall always think of with pleasure and satisfaction); with him, I had an opportunity of seeing, by examination of bodies after death, precisely what I had heard stated in the lecture-room, viz. a turgescence of arterial blood covering the origin of nerves going to parts affected with irregular actions; the name of convulsion is so much better known than its nature and cause, as I hope to warrant warrant a public notation of such appearances, with a view of the subject being further investigated.

The first case I witnessed (which will serve to elucidate my meaning) was a child about two years of age, a little patient of Dr. Sanders's, and I am sure he will allow me to refer to my notes upon this occasion for a brief detail of it to the public. The little sufferer had just recovered from the measles, when (which is not an uncommon occurrence,) he was seized with some affection of the mucous membranes of the lungs and bronchiæ, the respiration was difficult, the pulse quick and feeble, and asthma seemed a predominant symptom, the abdominal viscera seemed much deranged in their functions, to which succeeded spasms and convulsions. The muscles of both arms were acting irregularly, also the muscles of the eyelids and diaphragm; these actions alternated with crying, till that last repose which ends all our sufferings closed the scene: a short time prior to death, there appeared insensibility of both retinas.

*Dissection.*—I think it right here to state Dr. Sanders's prediction during the time the body was on the table for examination, which, at that time, struck my attention very forcibly: he observed, he did not expect to find any disease in the lungs or bowels sufficient to account for the fatal event, or even the symptoms which occurred during life; but that we should find effusion in the ventricles as well as at the base of the brain, round the tuber annulare, medulla oblongata, and top of the spinal canal at the foramen magnum, and that we should find arterial turgescence on the offset or visible origin of the nerves which served the convulsed organs. Upon opening the thorax, pus was found in the bronchial glands, and the lungs not sufficiently diseased to destroy life. In the brain, there was an increase of water in the lateral ventricles, also effusion in the fourth ventricle, and at the base; the plexus choroides was of a venal dark colour. On examining the nerves of the brain, both optics had a turgescence of red blood covering them at their origin for the space of an inch, appearing as if a brush, connected with red paint, had been drawn over them; or a better analogy will be to say, as if the small vessels of the nerves had been injected with subtle red wax, if such had been possible; the fourth pair of nerves had the same red appearance, also the eighth; and, on that part of the spinal marrow from whence the nerves of the anus originate, the vessels appeared dilated with red blood, perhaps to the utmost power of dilatability, shewing the existence of pure inflammation.

I have here done no more than noted facts, and will leave to the speculative mind to imagine the effects such alteration in in the structure of parts so pre-eminent in the functions of life is likely to produce.

In the prosecution of morbid anatomy, it must be the object first to ascertain what change takes place in the first alteration or disease; 2ndly, how that disease is communicated to other parts; and, 3rdly, how such diseases are likely to be relieved by our first knowledge. A learned author has said, that the knowledge of morbid structure does not lead with certainty to the knowledge of morbid actions, although the one is the effect of the other; yet, surely it lays the most solid foundation for prosecuting such enquiries with success: in proportion, therefore, as we shall become acquainted with the changes produced in the structure of parts from diseased actions, we shall be more likely to make some progress towards a knowledge of the actions themselves, although it must be very slowly.

It appears, then, that the knowledge of such diseases is useful in proportion as it directs us how to relieve the symptoms when they occur, which appears to me best done by the abstraction of blood from as near the origin of the nerve as possible, to relieve turgescence; then the application of a stimulus to make the vessels contract to their natural size, which will facilitate the operation of nature to a return of health.

I could here relate other cases, similar to the foregoing one, where effusion at the origin of nerves produced spasmodic actions in the parts supplied by the nerves thus affected; but such would be unnecessary, as I wish at present briefly to notice what I have seen on dissection, without entering into any physiological discussion of the cause, because Dr. Sanders has recently informed me, he is preparing a detailed and practical work, founded solely on Anatomical Examination, to shew the influence the whole contents of the head, and spinal canal, as well as all the nerves of the brain, tuber annulare, medulla oblongata, and spinal marrow, have individually and conjointly in the phenomena of disease; indeed, I understand, he has, for some time past, directed much of his leisure time to this subject, and I make no doubt the result will contribute largely to reduce the practice of medicine to simple and fixed principles. That he may speedily complete his work is the sincere wish of his friend; to work in such a cause is to work in the cause of humanity; it is infinitely more agreeable to save a single life than to have the command of an empire.

*Hereford;* P. P. JAMES, Surgeon.

*December* 22, 1814.

